# Supplementary material for: Accessing the In Vivo Efficiency of Clinically Isolated Phages against Uropathogenic and Invasive Biofilm-Forming Escherichia coli Strains for Phage Therapy
Source: Cells. 2023 Jan 17;12(3):344. doi: 10.3390/cells12030344 (PMC9913540; doi:10.3390/cells12030344)
Supplement: Supplementary file 1 [file cells-12-00344-s001.zip › cells-2083777-supplementary .pdf]

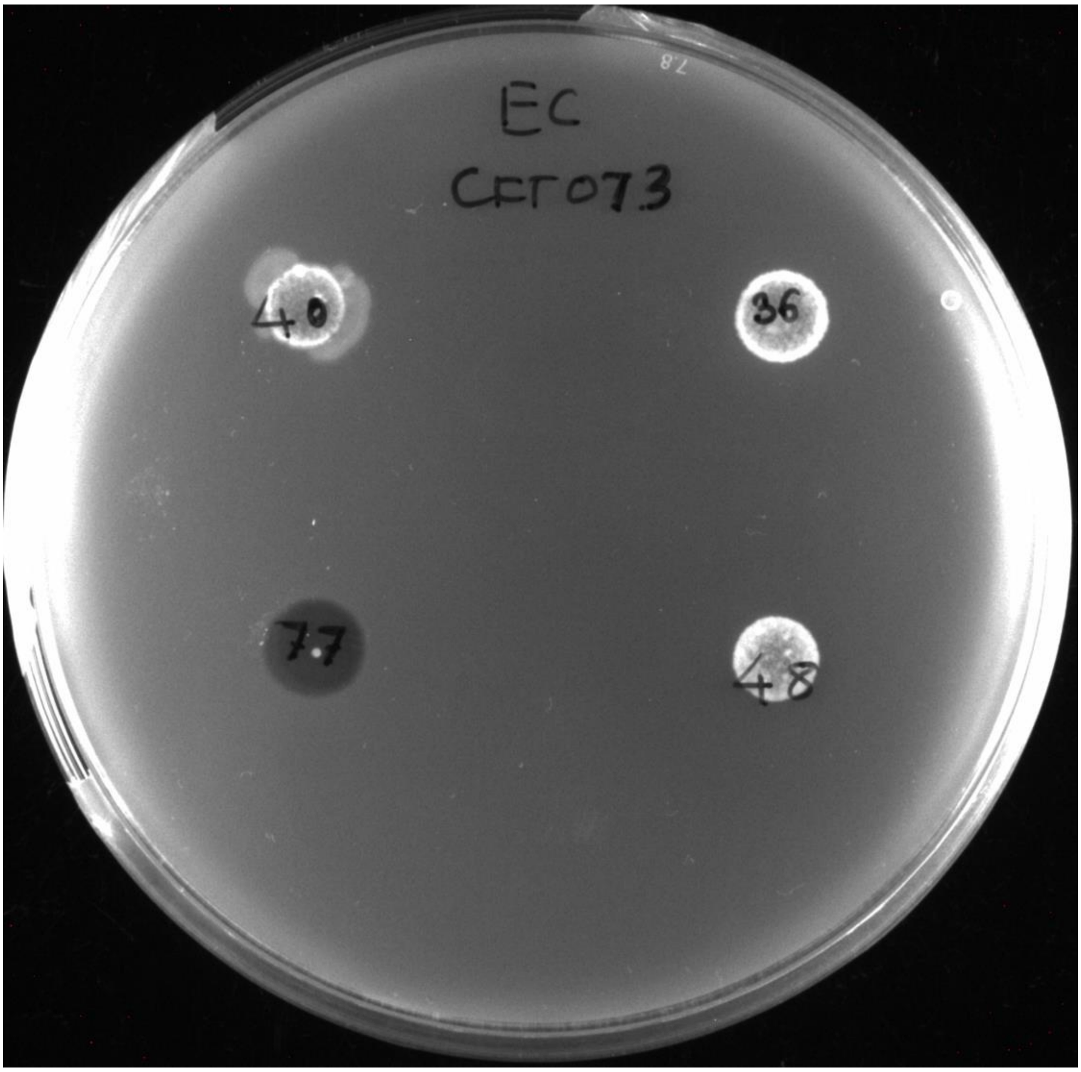

Figure S1. The cross-infectivity of selected clinically isolated phages against *E. coli* CFT073 biofilm-producing strain used for *in vitro* and *in vivo* studies in *Galleria mellonella*. Only IBEC77 shows lytic activity against the *E. coli* CFT073 strain.

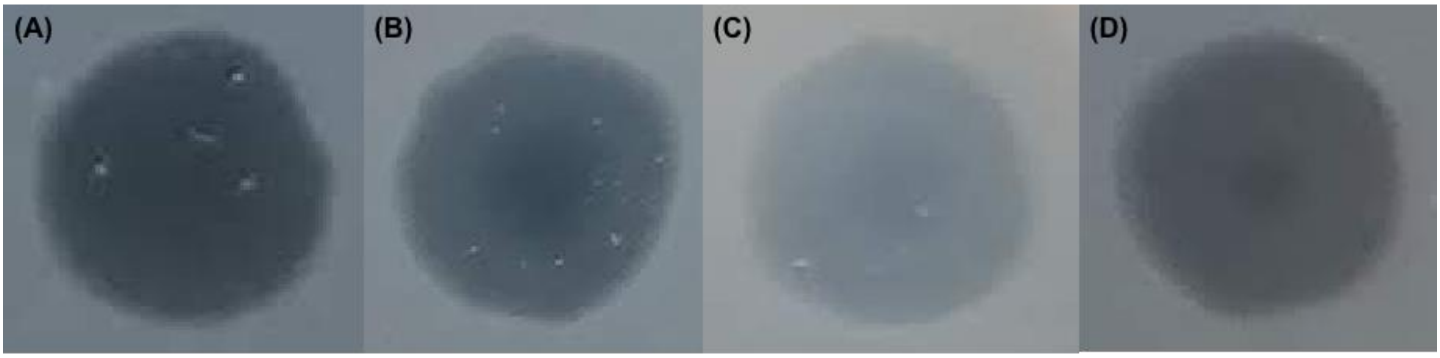

Figure S2. The plaque morphology of (A) IBEC 36, (B) IBEC 40, (C) IBEC 48, and (D) IBEC 77 isolated from clinical samples against *E. coli* LF82 (IBEC 36), *E. coli* MG1655 (IBEC 40 and IBEC 48), *E. coli* CFT073 (IBEC 77) biofilm-producing strain and used for *in vitro* and *in vivo* studies in *Galleria mellonella*.

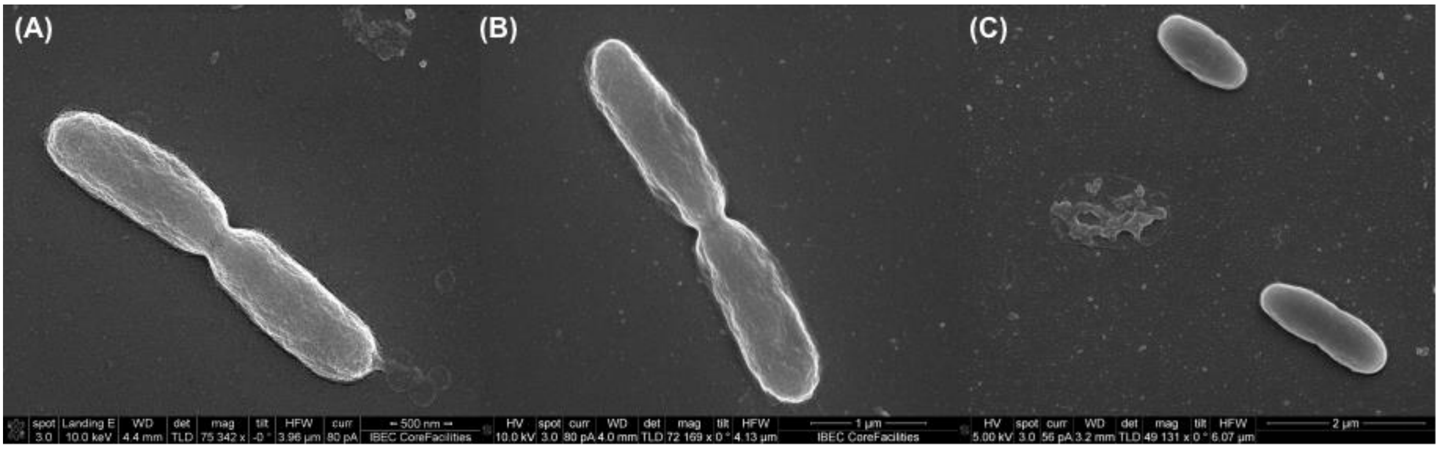

Figure S3. The control for scanning electron microscopy for phage uninfected (A) *E. coli* LF82, (B) *E. coli* MG1655, and (C) *E. coli* CFT073 biofilm-producing strain.
